# The Adolescent Depression Rating Scale (ADRS): a validation study

**DOI:** 10.1186/1471-244X-7-2

**Published:** 2007-01-12

**Authors:** Anne Revah-Levy, Boris Birmaher, Isabelle Gasquet, Bruno Falissard

**Affiliations:** 1 INSERM U669, Université Paris Sud 11, Centre Hospitalier V. Dupouy, 95107 Argenteuil, France; 2 Department of Child Psychiatry, University of Pittsburgh Medical Center, Western Psychiatric Institute and Clinic, Pittsburgh, USA; 3 INSERM U669, Université Paris-sud 11, APHP, Villejuif, F-94804, France

## Abstract

**Background:**

To examine the psychometric properties of the Adolescent Depression Rating Scale (ADRS), a new measure was specifically designed to evaluate adolescent depression.

**Methods:**

The 11-item clinician-report and 44-item self-report versions of the ADRS were developed from a qualitative phase involving interviews of experts and adolescents. These two instruments were then administered to 402 French speaking adolescents with and without depressive disorders. Item distribution, internal consistency, convergent validity, discriminant validity and factorial structure were assessed.

**Results:**

After reduction procedures, a 10-item clinician version and a 10-item self-report version were obtained. The ADRS demonstrated good internal consistency (alpha Cronbach coefficient >.70). It also discriminated better between adolescents with and without depression than the Hamilton Depressive Rating Scale and the Beck Depression Inventory (BDI-13).

**Conclusion:**

The ADRS is a useful, short, clinician-report and self-report scale to evaluate adolescent depression. Further studies to replicate our findings and evaluate ADRS sensitivity to effects of treatment and psychometric properties in populations of adolescents with several psychiatric disorders are warranted.

## Background

Standardized diagnostic criteria for depression have raised several questions regarding the classification of adolescents with depression. In fact, dichotomizing the population into cases and non-cases does not take into account that a substantial proportion of depressed adolescents have sub-syndromal depressive symptomatology [1]. Yet these young people have significant morbidity and are at high risk of developing full-blown major depression [2].

Recent findings [4,24] support the view that measures of depression in adolescence are best implemented in a dimensional model, where depressive disorders are conceptualized as a continuum of severity, from mild to severe. Thus, subjects who meet DSM-IV diagnostic criteria for MDD [3] represent the extreme of a continuum rather than a distinct group [4].

The feasibility of clinical trials in adolescent populations depends on the availability of valid measures [5]. In fact, many studies have utilized depression scales despite limited data regarding their validity with young people. For adolescents aged 13 and older, the most widely used self-reports include the Centre for Epidemiological Studies Depression Scale (CES-D) [6] and the Beck Depression Inventory (BDI) [7]. Likewise, the most frequently used clinician reports include the Hamilton Depression Rating Scale (HDRS) [8,9], the Montgomery-Asberg Depression Rating Scale (MADRS) [10], and the Children's Depression Rating Scale CDRS-R [11]. However, these instruments are not specific to adolescents, lack construct validity, and have limited or unknown reliability and validity in this age group [12-14].

Following the dimensional perspective outlined by Fergusson, the main aim of our study was to design and validate a dimensional scale, the Adolescent Depression Rating Scale (ADRS) to quantify the intensity of depression in adolescents aged from 13 to 20 years. On the basis of recommendations by Myers and Winters [13,14] (construct validity as the core of depression rating scale development, the use of two scales – self report and clinician versions – to obtain a robust measure, both having short forms to facilitate reiterated and longitudinal assessments), the decision was made to develop two versions of the ADRS: a self-report and a clinician-report instrument. The clinician-report ADRS is designed to be used in clinical and research settings and the self-report ADRS for epidemiological studies.

This paper reports on the validation of the French version of the ADRS. This validation is based on a multi-centre study in France, Belgium and Switzerland. A validation study is presently in progress for the English, Spanish, Chinese, Arabic, and Hebrew versions of the instrument.

## Methods

### Scale construction

The first phase was qualitative and designed for item generation and construction; it was based on four steps:

1. Apprehension of depression in adolescents via detailed exploration of the literature (Medline, PsycInfo, from June 1993 to June 2003, keywords: adolescent, depressive disorders, psychometrics, rating scales).

2. Qualitative studies on research interviews of 11 experienced clinicians in adolescent psychiatry (from 15 to 25 years of experience in adolescent psychiatry) and 5 depressed adolescents, to identify facets of adolescent depression (question grid in Annex, see additional file 1). The task was 1/to apprehend psychiatrists' representations of depressive disorders in adolescents, 2/to get an idea of experiences and practice, 3/to describe decisional steps for each clinician, 4/to apprehend in as neutral a manner as possible the symptomatic expression of the depressive experience, from the adolescent patient verbatim. These qualitative data were analyzed using an adaptation of Colaizzi's method [15], pinpointing the categories of depressive experience that emerged from the interviews. Features identified were grouped into themes, and then into theme clusters with common traits, and finally into more general categories. Depression in adolescents appeared as *a complex emotional state*, involving irritability, a feeling of being overwhelmed by the depressive experience, negative perceptions of self, thoughts about death; it was also found to involve *non-emotional manifestations*: mental slowness, sleep disturbances, and *manifestations through social interactions *at school, in work, in leisure, and in relationships with others.

3. Construction of the measure on the basis of the two previous steps: drafting of the items, and selection of items using a consensus method.

The ADSR was constructed using the three domains derived from the qualitative phase : 1) emotional state involving irritability, a feeling of being overwhelmed by the depressive experience, negative perceptions of self, and thoughts about death; 2) non-emotional manifestations involving mental slowing, and sleep disturbances; 3) clinical manifestations through social interactions at school, in work and leisure, and in relationships with others. Two initial versions of the ADRS were constructed: 1) an 11-item clinician-report scale, the ADRS initial version for clinicians (ADRSic) with depression severity rated on a 7-point Likert scale from none to severe, for the previous two weeks; 2) a 44-item self-report scale, the ADRS initial version for patients (ADRSip) with true/false responses (Table 1).

**Table 1:** French initial version of ADRS, patient version (ADRSip)

1.	J'arrive à rien en ce moment
2.	Je me laisse énerver pour un rien
3.	Au fond je ne me sens pas à la hauteur
4.	Je n'aime rien en ce moment
5.	Mes idées sont en désordre
6.	Je me perds dans le travail en ce moment, mais ça ne donne rien
7.	Je n'ai pas d'énergie pour l'école, pour le travail
8.	Je veux rester couché et dormir
9.	Au moins dans mon lit, je ne pense plus à rien
10.	J'ai du mal à réfléchir
11.	Je sens que la tristesse, le cafard me débordent en ce moment
12.	Je n'ai plus de plaisir à faire mes activités comme avant
13.	Penser, réfléchir, lire ou travailler me demande un effort
14.	Je fais des rêves horribles
15.	J'ai peur que ce que tout ça devienne insupportable
16.	Je sens bien qu'il vaut mieux que je ne sois pas seul(e)
17.	Il n'y a rien qui m'intéresse, plus rien qui m'amuse.
18.	J'ai même du mal à supporter mes copains
19.	Ce que je fais ne sert à rien
20.	J'en peux plus de ma vie en ce moment
21.	En ce moment je ne maîtrise pas ce qui se passe
22.	L'école, le travail, ça m'intéresse pas en ce moment, j'y arrive pas
23.	Je ne supporte rien
24.	J'ai du mal à rassembler mes idées
25.	Je ne supporte pas grand monde
26.	Je peux rester couché des heures à rien faire
27.	Je me sens seul à l'intérieur de moi
28.	Au fond, quand c'est comme ça, j'ai envie de mourir
29.	Je n'arrive pas à me concentrer
30.	Je trouve que rien ne vaut la peine en ce moment
31.	Je vois bien que je dois me forcer pour l'école, le travail
32.	Avec mes amis au moins je ne pense à rien
33.	Je ne supporte pas grand chose
34.	Ce que je fais ne vaut pas grand chose
35.	Je veux absolument pas être tout(e) seule, sinon je sens que ça va pas
36.	Je me sens découragé
37.	Je trouve tout trop difficile
38.	Ces derniers temps, j'ai arrêté mes loisirs, ça me dit plus rien
39.	On m'énerve facilement
40.	Je dors très mal
41.	Je veux rester chez moi et rien faire
42.	Je me sens dépassé par ce qui m'arrive
43.	A l'école, au boulot, j'y arrive pas
44.	Je vais pas pouvoir supporter tout ça longtemps

The modality of response was 'oui/non' (yes/no) for each item

An instruction manual was drafted for the ADRSic (Annex, see additional file 1).

### Validation

#### Sample and instruments

Four-hundred and nine adolescents, aged 13–20 years, who attended outpatient clinics or were hospitalized between November and May 2004 at 15 medical centres in France, in Brussels (Belgium) and in Geneva (Switzerland) were included. The patients recruited were all receiving treatment but not necessarily for depression: they were attending either a psychiatric clinic or an adolescent medicine clinic (80%; 20%).

Subjects with mental retardation or psychosis were excluded. To widen our data to subjects with different levels of depression, care was taken to include a population with a wide range of disorders and different levels of depressive severity, from none to severe.

All investigators were senior child and adolescent psychiatrists who were trained and supervised by the principal investigator (ARL) to administer the ADRSic. During the first interview, subjects and their parents were introduced to the nature of the study and asked to sign the consent form. Since this validation study was only observational, according to French law no authorization from an ethics committee was necessary.

All adolescents completed the BDI 13-item version [7] and the ADRSip (44 items). The investigators completed the ADRSic (11 items), the HDRS 17-item version [8,9], and the Clinical Global Impression Scale (CGI) severity scale [16]. Following this, the investigators were asked to answer a question requiring a clinical judgment by the investigator: "Based on your experience, do you consider that this patient is depressed? Does he/she require treatment for this aspect of his/her psychopathology?" Finally, based on the clinical interview, the investigators completed the DSM-IV criteria for MDD.

The two variables DSM-IV criteria for MDD (Yes/No) and clinical judgment (Yes/No) were then used to classify patients in 3 groups:

1) No depression: the patients did not meet DSM-IV criteria for MDD and were not considered clinically depressed by the investigator.

2) Intermediate group: the patients did not meet the DSM-IV criteria for MDD, but they were considered clinically depressed according the clinical judgment. These patients were experiencing a form of depression other than MDD.

3) Depression: the patients met DSM-IV criteria for MDD and were judged clinically depressed.

As mentioned above, since we were interested in evaluating the ability of the ADRS to ascertain depressive symptomatology in different levels of depression, the analyses of our data were performed for these three groups.

#### Data analysis

First, an item analysis was carried out on the initial versions, with a close inspection of the distribution of each item to detect any floor or ceiling effect, or any item redundancy.

Internal consistency was measured by means of the Cronbach coefficient. Concurrent validity was assessed by Pearson correlations computed between the HDRS, CGI, BDI and the ADRSp and ADRSc scores after item reduction.

The factorial structure of the ADRS initial and final versions was studied with a maximum likelihood factor analysis with Varimax rotation. The number of factors was determined from the observation of the screeplot, eigenvalues greater than one, and from the clinical interpretability of factors [17,18].

Using multiple group confirmatory factor analysis, the stability of the structure of the ADRS final versions was studied in relation to age, gender, and depression groups.

To assess the optimal cut-off scores derived from the total score so as to discriminate between levels of depression, the receiver operator curve (ROC) method was used [19]. Sensitivity and specificity were computed.

The ability of the ADRS final versions to discriminate among the different diagnostic groups was explored by computing the effect sizes across the three groups of patients defined according to level of depression. HDRS, CGI, BDI and the ADRSc and ADRSp versions were compared on this basis. The pairwise statistical comparison of these effect sizes was performed using a bootstrap procedure [20].

The "minimum clinically relevant differences" in ADRSc and ADRSp scores were estimated from the slope coefficient of the linear regression of ADRS on CGI [21].

Statistical analyses were performed using the R 2.0 package with "psy" and "mva" packages. Most psychometric analyses were replicated by a second statistician using SAS 8.2. Multiple group confirmatory factor analysis was performed with Mplus 2.1 [22].

## Results

### Sample

Four-hundred and two adolescents aged 16.5 (sd 2.0), 125 males (mean age 16.5 (sd 1.8)), 277 girls (mean age 16.6 (sd 1.9)) were assessed. One-hundred and twenty-six patients were depressed (mean age 16.4/sd 1.9; 32 males, 94 girls), 139 patients had a depressive experience but no MDD (mean age 16.6 (sd 1.8); 45 males; 94 girls) and 137 patients were not depressed (mean age 16.7 (sd2.1); 48 males, 89 girls). Seven patients out of 409 patients initially included were dropped because of missing data.

### Item analysis

For both clinician and self-report initial versions of the ADRS, the proportion of missing data per item was low (<5%). There was no item responses with floor effect >50%, or ceiling effect >50%. Inter-item correlation was <0.70, meaning there was no redundancy.

### Factor structure

For the initial version of the clinician-report ADRS over the whole sample of patients, the screeplot is in favour of a uni-dimensional instrument, since 44% of variance is contained in the first principal component while a second eigenvalue is approximately equal to 1 (1.05). This justifies the use of a single score as a summation of all items. To explore structure more precisely a factor analysis was conducted for a 2-factor solutions. This solution was clinically interpretable, with a factor that could be related to an "internal negative state" including irritability, feelings of being overwhelmed, negative perception of self, ideas of death, sleep disturbances and the empathetic perceptions of the clinician; and a second factor grouping "external manifestations" (including school, leisure activities and relationships). Most items were clearly attributable to a single factor except for the item "clinging relationships". Since this item was sometimes found difficult to rate by some investigators, it was discarded. On the remaining 10-item ADRSc (table 3), a new series of factor analyses was performed across the 3 groups defined according to a level of depression (table 4).

**Table 3:** English final version of ADRS clinician version (ADRSc)

**Item #**	**Item title**	**Modality of response**	**points**
1	Irritability	Absence of any irritability, either self-perceived or perceived by the observer	0
		Irritability perceptible or felt in dealings with the subject, although he/she can control it	2
		Considerable irritability in dealings with the subject, generating conflict, relational difficulties	4
		Intense, overpowering irritability making dealings and exchanges virtually impossible	6
2	Overwhelming experience of depression	No depressive feelings or thoughts (= gloominess, despair, sadness)	0
		Depressive feelings or thoughts present but controlled and manageable	2
		Feeling of being overwhelmed by depressive feelings or thoughts	4
		Intense feeling of being overwhelmed by depressive suffering that is devastating and impossible to contain	6
3	Negative perceptions of self	Perceptions of self are serene and relevant	0
		Tendency to depreciation of self and accomplishments	2
		Depreciation of self and accomplishments	4
		Self viewed as completely worthless, useless, overpowering despair	6
4	Ideas of death	No preoccupation regarding death or suicide	0
		Occasional preoccupations regarding death or suicide	2
		Recurrent preoccupations regarding death or suicide	4
		Pervasive and intrusive ideas regarding death or suicide	6
5	Mental slowing	No sign of mental slowness, thought and speech fluid	0
		Occasional difficulties in putting ideas together, mental inertia that hinders concentration	2
		Considerable difficulty concentrating, obvious repercussions on daily life or school	4
		Massive mental inertia, that can result in concentration being impossible or the interview being difficult	6
6	Sleep	No sleep disturbance, whether in duration or quality(= sleeplessness, nightmares, not feeling rested, sleeping excessively)	0
		Occasional sleep disturbance, unusual to the subject	2
		Marked, persistent sleep disturbance	4
		Major, persistent sleep disturbance, resistant insomnia	6
7	Investment in school, work or job seeking	Sustained investment in school or professional activities	0
		Loss of motivation for school or work, but activities maintained	2
		Marked loss of motivation, disinterest for school or professional activities	4
		Total loss of motivation, complete disinterest for school or professional activities	6
8	Investment in non-school activities	Interest and enjoyment intact, good investment in usual non-school activities	0
		Decrease of enjoyment or interest in usual non-school activities, but these are nonetheless maintained	2
		Loss of enjoyment or interest, repeated absence from usual activities, marked narrowing of activities	4
		Absence of enjoyment or interest in non-school activities, total cessation of usual activities	6
9	Relationship withdrawal	No relational withdrawal	0
		Unusual withdrawal from others	2
		Relational withdrawal, isolation from others	4
		Total isolation	6
10	Perceived empathy from the clinician	Interview felt to have occurred in a serene atmosphere	0
		Perception of sadness pervading the interview	2
		Feeling there was over-riding emotion and/or irrepressible sadness	4
		Perception of intense silent distress	6

Intermediate responses are allowed from 0 to 6.

One item has been removed from the initial version: "clinging relationships".

**Table 4:** Maximum Likelihood Factor Analysis of the ADRSc in three groups

	**Not depressed (n = 137)**		**Depressed according to clinician but not to DSM-IV (n = 139)**		**Depressed according to clinician and DSM-IV (n = 126)**	
	**Factor 1**	**Factor 2**	**Factor 1**	**Factor 2**	**Factor 1**	**Factor 2**

Irritability	0.997	-0.031	0.380	-0.020	0.166	0.038
Overwhelming depression	0.340	0.507	0.797	0.168	0.653	0.261
Negative perception of self	0.303	0.209	0.468	0.177	0.343	0.289
Ideas of death	0.314	0.130	0.424	0.095	0.670	0.098
Mental slowness	0.156	0.594	0.208	0.371	0.283	0.292
Sleep	0.222	0.212	0.273	0.068	0.412	0.247
School investment	0.145	0.422	0.149	0.455	0.272	0.629
Leisure	0.137	0.476	0.077	0.919	0.103	0.887
Relationships	0.061	0.488	0.046	0.542	0.277	0.624
Empathetic perception of clinician	0.518	0.487	0.741	0.258	0.658	0.385

ADRSc: Adolescent Depression Rating Scale Clinician version

The stability of the two-factor solution was tested across age (under 16 or 16 and over), gender, and level of depression. There was no gender effect on the structure of the two versions of the ADRS. Factor loadings were however statistically significantly different across age and depression groups (all p values < 0.05).

Overall, the results are similar for the initial patient-report ADRS, especially with regard to the structure of the instrument. However, to be useful in epidemiological studies a self-report measure should be of a limited size. Therefore an item reduction procedure was carried out on the initial 44-item self-report. Items were removed on the basis of clinical considerations (clinical importance of the item itself), but also on the basis of readability, relevance, redundancy, item distribution across the different domains of the depressive experience derived from the qualitative phase, and statistical considerations (loading < 0.4, inter-item correlation > 0.6).

This procedure yielded a 10-item instrument, ADRSp (table 2). A factor analysis conducted on this shorter instrument confirmed a two-factor structure comparable to the structure of the clinician version reported above (table 5). We present the Factor analysis of the final ADRSc and ADRSp for the three groups of the sample.

**Table 2:** French and English final version of ADRS patient version (ADRSp)

**Item #**	**English version**	**French version**
1	I have no energy for work/school	Je n'ai pas d'énergie pour l'école, pour le travail
2	I have trouble thinking	J'ai du mal à réfléchir
3	I feel overwhelmed by sadness and listlessness	Je sens que la tristesse, le cafard me débordent en ce moment
4	Nothing really interests or entertains me	Il n'y a rien qui m'intéresse, plus rien qui m'amuse.
5	What I do is useless	Ce que je fais ne sert à rien
6	When I feel this way I wish I were dead	Au fond, quand c'est comme ça, j'ai envie de mourir
7	Everything annoys me	Je ne supporte pas grand-chose
8	I feel downhearted and discouraged	Je me sens découragé
9	I sleep badly	Je dors très mal
10	School/work doesn't interest me just now, I can't cope.	A l'école, au boulot, j'y arrive pas

The items selected from the initial versions are items # 7,10,11,17,18,28,33,36, 40 and 43.

The modality of response was 'vrai/faux' (true/false) for each item.

**Table 5:** Maximum Likelihood Factor Analysis of the ADRSp in three groups

	**Not depressed (n = 137)**		**Depressed according to clinician but not to DSM-IV (n = 139)**		**Depressed according to clinician and DSM-IV (n = 126)**	
	**Factor 1**	**Factor 2**	**Factor 1**	**Factor 2**	**Factor 1**	**Factor 2**

No energy for work	0.041	0.997	0.125	0.990	-0.014	0.712
Trouble thinking	0.547	0.147	0.246	0.032	0.289	0.344
Overwhelmed by sadness	0.597	0.247	0.613	0.069	0.570	0.190
Nothing really interests me...	0.665	0.107	0.389	0.142	0.579	0.225
What I do is useless	0.413	0.128	0.368	0.193	0.532	-0.001
Ideas of death	0.572	0.175	0.474	0.098	0.614	-0.069
Everything annoys me	0.628	0.046	0.582	0.157	0.474	0.094
Discouraged	0.552	0.207	0.559	0.344	0.507	0.057
Sleep badly	0.225	0.211	0.448	0107	0.599	0.092
Not coping at school/work	0.229	0.510	0.166	0.538	0.076	0.794

ADRSp: Adolescent Depression Rating Scale patient version

### Scale internal consistency

The internal consistency of the two final versions of the ADRS was good. The Cronbach alpha coefficient for ADRSp was 0.74 in the non-depressed group; 0.74 for the intermediate group and 0.79 for depression group. For the ADRSc, the Cronbach alpha was 0.78 in the non-depressed group, 0.75 for the intermediate group and 0.75 for the depression group

### Concurrent validity

All instruments were significantly correlated. As shown in table 6, Pearson correlation coefficients between ADRSc and the HDRS, BDI and the CGI ranged from 0.62 to 0.8 (all p-values < 0.05). Pearson correlations between the ADRSp and the HDRS, BDI and the CGI ranged from 0.51 to 0.83 (all p-values < 0.05).

**Table 6:** Pearson's correlations coefficient between ADRS, HDRS, BDI and CGIs

	**ADRSc**	**ADRSp**	**HDRS**	**BDI**	**CGI**
**ADRSc**	-	0.63	0.80	0.61	0.80
**ADRSp**		-	0.56	0.82	0.52
**HDRS**			-	0.52	0.74
**BDI**				-	0.48
**CGI**					-

all p-values < 0.05

ADRSc: Adolescent Depression Rating Scale (clinician version)

HDRS: Hamilton Depression Rating Scale

ADRSp: Adolescent Depression Rating Scale (self-rated version)

BDI: Beck Depression Inventory

CGI: Clinical Global Impression Severity Scale

It is noteworthy that the CGI ratings are more strongly correlated with the ADRS (self or clinician-rated) than with the HDRS or the BDI-13 (statistically significant with a bootstrap procedure).

### Discriminant validity: contrasted groups

As shown in Table 7, effect sizes of the HDRS, BDI, ADRSc and ADRSp were compared among the three groups. These effect sizes are intended to assess the sensitivity of the ADRS in differentiating groups with and without depression. The ADRSp shows a larger effect size than the BDI. This difference was statistically significant at the 5% level in differentiation of the non-depressed and depressed groups (including intermediate group). The ADRSc shows a larger effect size than the HDRS, and this difference was statistically significant in differentiating the groups [no depression] and [intermediate depression + depression] and in differentiating the groups [no depression + intermediate depression] and [depression].

**Table 7:** Discriminant validity and Minimum clinically relevant difference of ADRSc, ADRSp, HDRS and BDI

	**ADRSc**	**HDRS**	**ADRSp**	**BDI**
	
	**mean (sd)**	**mean (sd)**	**mean (sd)**	**mean (sd)**
	
Levels of depression				
No depression	8.9 (6.1)	6.0 (4.2)	2.3 (2.5)	8.6 (6.8)
Intermediate group	17.7 (7.6)	11.0 (5.4)	4.1 (2.7)	12.9 (8.3)
Depression	28.2 (8.9)	17.2 (6.2)	6.0 (2.7)	19.4 (8.9)
Effect size				
no dep *vs *(inter+dep)	1.41	1.21	0.94	0.79
(no dep+inter) *vs *dep	1.51	1.30	1.01	0.94
Sensitivity/specificity	Cut-off 15: 0.76/0.80 Cut-off 20: 0.83/0.78		Cut off 3: 0.79/0.60 Cut-off 4: 0.80/0.60	
Minimum clinically relevant difference	5.2	3.1	0.9	2.5

ADRSc: Adolescent Depression Rating Scale (clinician version)

HDRS: Hamilton Depression Rating Scale

ADRSp: Adolescent Depression Rating Scale (self-rated version)

BDI: Beck Depression Inventory

No depression: the patients did not meet DSM-IV criteria for MDD and were not judged clinically depressed by the investigator.

Intermediate depression group: the patients did not met the DSM-IV criteria for MDD but were judged clinically depressed.

Depression: the patients met DSM-IV criteria for MDD and were judged clinically depressed.

### Receiver operator curve analysis: sensitivity and specificity

The optimal cut-off point was determined by plotting sensitivity versus specificity for each possible cut-off and examining the point that maximized the summation of sensitivity and specificity. Here, two binary "gold standards" can be proposed to compute sensitivity and specificity: depression according the clinical judgment, or depression according to both clinical judgment and DSM-IV. Both analyses were performed. For the ADRSp, the cut-off corresponding to the clinical judgment of depression was 3 and the cut-off corresponding to the DSM-IV was 4. For the ADRSc, these cut-offs were 15 and 20, respectively. Sensitivity and specificity are reported in table 7.

### Minimum clinically relevant difference

Finally, minimum clinically relevant variations in score on a scale are of major interest when interpreting results of clinical trials or epidemiological studies. This minimum variation can be estimated from the slope coefficient of the linear regression of ADRS on CGI (Norman et al. 2001) (Table 7). As a rule-of-thumb, a variation of 5 points on the ADRSc may be considered as the minimum clinically important difference, while a variation of 1 point is clinically important for the ADRSp.

## Discussions and conclusions

The purpose of this study was to develop and validate a depression scale especially designed for adolescents. Our study showed that both the clinician and self-report versions of the ADRS had acceptable psychometric properties with good convergent, discriminant, and factorial validity, and good internal consistency.

The final 10-item versions of the clinician-report and self-report ADRS yielded two factors: "internal state" (items: irritability, overwhelmed by depression, negative perceptions of self, ideas of death, sleep and "external manifestations" (mental slowness, implication in school, leisure activities, relationships) (table 2). The factorial structure of the ADRS reinforces the construct validity of the scale since it is consistent with the preliminary qualitative phases of the development the ADRS scales. Depression in adolescents did indeed appear as *a complex emotional state*, involving irritability, a feeling of being overwhelmed by the depressive experience, negative perceptions of self, thoughts about death; this is accompanied by *non-emotional manifestations*: mental slowness, sleep disturbances, and *manifestations through social interactions *at school, work, in leisure, and in relationships with others.

From the psychometric point of view, the non-emotional manifestations are divided into two types: one is linked to the complex emotional state and forms the dimension "Internal state", the other is linked to manifestations through social interactions and forms the dimension "External manifestations". Internal state and external manifestations are dimensions that are already well known in depression, and they are described here in a way that is specific to adolescence. Although deficiencies in social relationships are not included in the definition of depression, any exploration of depression should place strong emphasis on the impact of social skill deficits and dysfunctional interpersonal behaviours [23].

It can be noted that in the ADRS the item "leisure" in the clinician version and the item "nothing really interests me or entertains me" in the self-report version explore manifestations of anhedonia, and the item "negative self-perception" explores one facet of a symptom often pointed to in depression: feelings of worthlessness. Recently, Wilcox and Anthony [24]) reported that persistent anhedonia during childhood or adolescence-was 17 times more prevalent in a sample of male adults with major depressive disorder than in a sample of controls, and this figure increased to 31–32 times in females. Feelings of worthlessness in childhood and adolescence were also associated with major depressive disorder in adulthood. Pine [25] and Murphy [26], also pointed to the prognostic significance of persistent anhedonia when it appears before early adulthood.

From a public health standpoint, identifying specific early clinical features might make it possible to provide anticipatory guidance or other early interventions that could prevent or reduce the impact of depressive disorders. In addition, the small number of items in the ADRS self-report involves little respondent burden, thereby allowing it to be combined with other short instruments that screen for different mental health conditions, enabling its use in epidemiological studies.

The results of this study need to be considered in light of the following limitations. First, only five adolescents were interviewed in the qualitative phase, this may be considered as a small number, even if a saturation of the *verbatim *collected appeared at this stage. Second, the sample under study was made up of psychiatric and medical outpatients or inpatients at multiple sites, even if this heterogeneity may be interesting in terms of generalisability, it may be criticised on scientific grounds. The strategy used to classify patients in the sample needs also to be discussed. A dimensional scale must be able to assess different levels of depression ranging from none to severe, for instance in clinical trials before and after treatment. Thus in our sample, three groups of depressive experience were formed: the non-depressed group, the intermediate group comprising patients who were depressed but not MDD (without consideration of the categories of depression), and the MDD group. We took the MDD form as the most severe form of depressive experience [4,24].

Other limitations must be noted. Inter-rater agreement was not measured in this first validation study. Also, since there is substantial overlap between anxiety and depressive symptomatology, subsequent studies should evaluate whether the ADRS differentiates between young people suffering from anxiety alone and those with depression [27]. These two points will be studied in a second validation step.

Finally, no standardized instruments were used to assess subjects' psychopathology. However, all interviewers were senior psychiatrists with vast experience in assessing and treating children with psychiatric disorders. In addition, to diagnose MDD they used the DSM-IV criteria. Perhaps a replication study could implement standardized strategies to classify the patients according to depression categories and to explore co-morbidities.

The ADRSp and ADRSc have been designed and validated in French (in France, Belgium and Switzerland). Translations have been made into English (Canadian, American, British), Spanish, Italian, Arabic and Hebrew. Back-translations have been performed for all these versions, and problems remain with the Arabic and Hebrew versions of the ADRSc and the English version of the ADRSp.

## Competing interests

The author(s) declare that they have no competing interests.

## Authors' contributions

ARL: initiation of the research, study concept and design, performing statistical analyses, interpretation of data, drafting the manuscript. This study was conducted as a thesis project for ARL under the supervision of BF.

BB and IG: participation in the interpretation of the results, and revision of the draft paper

BF: supervision of the statistical analysis and revision of the draft paper

All authors read and approved the final manuscript.

## Pre-publication history

The pre-publication history for this paper can be accessed here:



## Supplementary Material

Additional File 1 Annex. The text provided comprises: A – Question grid: list of the questions used for semi-structured interview of the qualitative phase (patient and clinician version). B – Interview guide of the ADRSc (clinician version) Click here for file
