# Supplementary material for: The Adolescent Depression Rating Scale (ADRS): a validation study
Source: BMC Psychiatry. 2007 Jan 12;7:2. doi: 10.1186/1471-244X-7-2 (PMC1785378; doi:10.1186/1471-244X-7-2)
Supplement: Additional File 1 — Annex. The text provided comprises: A – Question grid: list of the questions used for semi-structured interview of the qualitative phase (patient and clinician version). B – Interview guide of the ADRSc (clinician version) [file 1471-244X-7-2-S1.doc]

**Annex**

**A- Question grids: list of the questions used for semi-structured interview of the qualitative phase**

*Qualitative interviews with patients*

1/ Could you describe the period before you meet a doctor, or psychiatrist?

2/ What were your feelings during the initial period?

3/ How could you describe your relations to your family, to your friends?

4/ What about your time in school, and your school work?

5/ How did you realize what was going on for you?

6/ If you had to explain to some one what happened to you, how would you say it?

7/ Could you recognize one of your friend living the same experience? Based on what kind of signs, words, and sentences?

8/ When you met the psychiatrist at the beginning, do you remember what you wanted to explain to him/her?

*Qualitative interviews with psychiatrist*

1/ Can you describe the different expressions of adolescent depression?

2/ What are the main difference with adult depression and child depression?

3/ What are the significant symptoms related to the intensity of the depressive disorders?

4/ Did you notice formulations, words used by the depressed adolescent to describe what is happening to him when he is depressed?

5/ How do you manage your interview with an adolescent looking for a current depressive experience?

6/ What do you think about the categorical classification in adolescent depressive disorders?

7/ What tools do you use in your clinical practice? In your research studies in adolescent depression?

8/ What are your criticises about this tools?

9/ What are you explaining to the young psychiatrists about adolescent depression?

**B- Interview guide of the ADRSc (clinician version)**

Adolescence is a period marked by change and transformation, soliciting mental processes dealing with loss and separation. One should distinguish between, on the one hand, the normally fluctuating emotional states central to the adolescence process, and, on the other, a clinical state of depression, indicating failure of this process.

Depression in adolescence is viewed here through three major aspects of the depressive experience:

I. **The emotional state**: irritability, feelings of being overwhelmed by the depressive experience, negative perception of self, thoughts about death,

II. **The neurovegetative state** : mental slowing, sleep disturbance,

III. **Changes in social interactions**: work, leisure activities, relationships (withdrawal or clinging).

IV. The last item aims to explore the **empathetic perception of the clinician** through the course of the interview with the adolescent.

###### I. Emotional state

**1. Irritability**

Irritability is at the centre of the depressive experience in adolescents. The irritable adolescent does not seem able to put up with much. This can appear in the form of marked reactivity, often dominant in exchanges with others. It is not the conflict that triggers irritability, but rather an irritable state which results in conflict with others (peers, family, teachers), making relationships stormy and wearisome. The irritability may be self-perceived, perceived only by those close to the adolescent, or perceived by outside observers. It may relate to all areas of activity or, at first, involve only the home setting.

**2. Overwhelming depressive experience**

The depressed adolescent is not always able to identify what he/she is feeling and cannot say whether it is sadness, fear or anxiety. He/she may feel in danger of losing control, or being overwhelmed by painful and/or unbearable experiences.

Gradual levels of severity might be:

- fear of losing control, but emotions remain contained
- feeling of being overwhelmed by the depressive experience
- over-riding feeling of being unable to control what is happening

**3. Negative perception of self**

The depressive experience affects self-perception. Depreciation of personal achievements may be prominent, leading the adolescent to lose confidence in his/her abilities, and his/her self-perception of being.

**4. Thoughts about death**

Themes of death and suicide are common in the existential questioning of adolescents. Thus, it is not the presence of these themes, per se, that indicates depression, but rather their pervasive character in the subject’s thoughts, death and suicide being viewed as the only way to escape current suffering. The clinician should be looking for possible thoughts of death behind the loss of meaning of daily activities, making the subject feel that nothing is worth the effort, nothing is worth doing or living.

**II. Neurovegetative state**

**1. Mental slowing**

This does not refer to withdrawal, shyness, or embarrassment upon meeting with the clinician, but to a slowing down or a form of inertia in the thought process, making the interview difficult. The adolescent visibly has to make an effort to give something thought or consideration. he/she has difficulty gathering and expressing ideas. He/she may confirm that concentration is difficult and thinking is slow. This slowing down may affect school work, which becomes extremely lengthy and inefficient, and may hinder reading.

**2. Sleep**

Sleep patterns normally vary in the course of development, with changes in duration and quality at the time of adolescence. The effect of depression is to disorganise the sleep and alter its quality: frequent awakenings, repeated nightmares, short or excessively long nights. The sleep disturbance has to be unusual for the subject, first occurring occasionally, then becoming persistent. In the absence of a sleep disorder, the excessive use of bed or sleep (e.g., wanting to stay in bed and/or asleep) may be rated 2: “Occasional sleep disturbance, unusual to the subject”

**III. Changes in social interactions**

The adolescent is in a process of transformation that makes him/her vulnerable to the outside, and generates questions regarding his/her place in the social world (investment in work, school, leisure), and his/her relationship with others. In the depressive experience, all social interactions are altered or hindered.

**1. Investment in school, work or job seeking**

Here, investment is assessed as regards the adolescent’s motivation for school or for work. Obviously, school and professional difficulties may result from various causes, and the clinician should differentiate loss of motivation for school/work, *to be rated in this item*, from difficulties linked to mental slowness and poor concentration, already rated above and not to be taken in consideration here.

**2. Investment in non school (leisure) activities**

Leisure activities, and the pleasure they provide, are a privileged area for adolescent self-development, aside from the family world. The depressed adolescent will withdraw from leisure activities, due to loss or decrease of interest and pleasure in occupations previously valued and regularly attended. Decreased interest and pleasure will first lead to irregular attendance, under the excuse of being tired or consumed by work, up to complete refusal. The adolescent may gradually cut down on activities, until he/she refuses to do anything, or lay sprawled watching TV, in an uncharacteristic manner for him/her.

**3. Relationships**

Relationships with others are central to the adolescent’s daily living. In the depressive state, changes in interpersonal relationships will manifest either the depressive withdrawal process (isolation) or the defence against it (clinging relationship with girlfriend, boyfriend or parental figure). Depressed adolescents may also seek multiple contacts, in order to forget and not think. Depressive “clinging” should be differentiated from the use of a contra-phobic figure in anxiety disorders, not to be rated here.

**IV. Empathetic perceptions on the part of the clinician**

This pertains to the subjective perception of the clinician from his/her encounter with the adolescent. In the course of the interview, empathy may enable the clinician to pinpoint cues of the adolescent’s depressive experience. The clinician will be sensitive to the atmosphere of the interview, to the degree of emotion present, the intensity of the unexpressed distress, beyond or outside the words actually used by the subject to describe the feelings he is experiencing. The clinician’s perceptions will enrich his/her clinical judgment.
